# The Association between Serum Cytokines and Damage to Large and Small Nerve Fibers in Diabetic Peripheral Neuropathy

**DOI:** 10.1155/2015/547834

**Published:** 2015-04-16

**Authors:** Francesca Magrinelli, Chiara Briani, Marcello Romano, Susanna Ruggero, Elisabetta Toffanin, Giuseppa Triolo, George Chummar Peter, Marialuigia Praitano, Matteo Francesco Lauriola, Giampietro Zanette, Stefano Tamburin

**Affiliations:** ^1^Department of Neurological and Movement Sciences, University of Verona, Piazzale Scuro 10, 37134 Verona, Italy; ^2^Department of Neurosciences, Sciences NPSRR, University of Padova, Via Giustiniani 5, 35128 Padova, Italy; ^3^Neurology Unit, Azienda Ospedaliera Ospedali Riuniti Villa Sofia Cervello, Piazzetta Salerno 3, 90146 Palermo, Italy; ^4^Internal Medicine Unit, Azienda Ospedaliera Ospedali Riuniti Villa Sofia Cervello, Piazzetta Salerno 3, 90146 Palermo, Italy; ^5^Diabetology Unit, Pederzoli Hospital, Via Monte Baldo 24, 37019 Peschiera del Garda, Italy; ^6^Neurology Unit, Pederzoli Hospital, Via Monte Baldo 24, 37019 Peschiera del Garda, Italy

## Abstract

Diabetic peripheral neuropathy (DPN) is a frequent complication of type 2 diabetes mellitus (DM) and may involve small and large peripheral nerve fibers. Recent evidence suggests a role of cytokines in DPN. The paper is aimed at exploring whether the serum concentration of cytokines is associated with small and large nerve fiber function and with neuropathic pain (NP). We recruited a group of 32 type 2 DM patients who underwent serum cytokines (TNF-*α*, IL-2, IL-4, IL-6, and IL-10) dosage as well as electrodiagnostic and quantitative sensory testing (QST) assessment to explore damage to large and small nerve fibers. Raised serum levels of IL-6 and IL-10 correlated with markers of large nerve fiber sensory and motor axonal damage. Raised IL-10 serum level was associated with signs of motor nerve demyelination. No differences were found in pain characteristics and electrodiagnostic and QST markers of small nerve fiber function in relation to cytokines serum levels. IL-6 and IL-10 serum levels were associated with large nerve fiber damage but not to small fibers function or NP. IL-6 and IL-10 cytokines might play a role in the pathogenesis of nerve fiber damage or represent a compensatory or neuroprotective mechanism.

## 1. Introduction

Diabetic peripheral neuropathy (DPN) is the most common long-term complication of type 2 diabetes mellitus (DM) and affects approximately half of the patients over the course of disease [1]. Small and large peripheral nerve fibers may be involved in DPN. Large nerve fiber damage causes paresthesia, sensory loss, and muscle weakness and small nerve fiber damage is associated with pain, anesthesia, foot ulcer, and autonomic symptoms. In clinical practice, the diagnosis of DPN is made on the base of questionnaires and clinical evaluation and electrodiagnostic studies are considered to be rarely helpful [1]. Nerve conduction study (NCS) however is important in experimental studies in that it may document the extent and severity of nerve involvement and demonstrates the presence of axonal damage and/or demyelination.

The mechanisms of DPN are only partially understood, and hyperglycemia, dyslipidemia, and insulin resistance are considered to be involved in its pathophysiology. The observation that successful treatment of hyperglycemia often fails to prevent DPN suggests the presence of early biochemical mediators that, once activated, may act independently of the initial stimulus. The biochemical pathways leading to nerve damage in DPN are complex and they include the accumulation of sorbitol, lipoxygenase activation, oxidization, and glycation of proteins and lipoproteins. Glucose and oxidized and glycated proteins may bind various receptors on neurons and endothelial cells and activate inflammatory signaling mechanisms and disrupt mitochondrial metabolism leading to oxidative stress and DPN [2].

Chronic hypoxia secondary to microvessel involvement and accumulation of advanced glycation end products [2, 3] may contribute to the induction of a low-grade chronic, subclinical inflammation in patients with DPN [4, 5]. Emerging evidence indicates that cytokines including tumor necrosis factor alpha (TNF-*α*), interleukins (ILs), adhesion molecules, chemokines, and other soluble molecules may be involved in the pathogenesis of experimental diabetic neuropathy [2]. Cytokines have been documented to contribute to an array of peripheral neuropathies, including immune-mediated neuropathies and chemotherapy-induced peripheral neuropathy [6].

Neuropathic pain (NP) is a common complication of DPN and is associated with peripheral and central mechanisms of sensitization [1, 7]. Cytokines have been found to contribute to the pathogenesis of NP in animal models [8] and in humans [9].

The main limitations of animal models of DPN and NP [10] are that they do not clearly separate DPN from NP and that they mainly explore evoked pain, whereas patients often complain of spontaneous NP [11, 12]. There are few reports on the role of cytokines in patients with DM, where the diagnosis of DPN was usually made on the basis of questionnaires and/or clinical evaluation [4, 13–15], but they leave some open questions, because neurophysiological or psychophysical data on the type and amount of peripheral nerve damage, as well as hints about possible mechanisms of nerve damage, are scarce.

To add a piece of information on this topic, the present study is aimed at exploring the possible role of cytokines as markers of peripheral nerve damage in DPN. To this goal, electrodiagnostic and quantitative sensory testing (QST) measures of loss-of-function of large and small nerve fibers were recorded in a group of patients with type 2 DM. NCS explores the function of large (A-beta) nerve fibers, while sympathetic skin response (SSR) documents the damage of small (myelinated A-delta and unmyelinated C) nerve fibers [16]. Reduced amplitude of sensory and motor potential indicates axonal damage and reduction of conduction velocity is associated with demyelination. F-wave latency measures the conduction across the whole nerve and is sensitive to changes at the proximal nerve site [17]. QST explores the presence of gain- and loss-of-function signs of different nerve fibers [18]. Among QST measures, we focused on thermal detection thresholds, which document damage to small nerve fibers. We correlated signs of damage to different nerve fibers to the presence and serum concentration of cytokines. We included type 2 DM patients regardless of the presence/absence of DPN because we were interested in the correlation between cytokine levels and nerve fiber function across different stages of DPN, including preclinical ones.

## 2. Materials and Methods

### 2.1. Subjects

We recruited 32 subjects (17 men, mean age 63.6 ± 9.3 years, range 47–79) who were affected with type 2 DM according to American Diabetes Association criteria [19] and fulfilled the following inclusion/exclusion criteria: age ≥ 18 years, no cognitive impairment (Mini Mental State Evaluation score ≥ 25/30), no coexistent neurological disease except for DPN, no severe systemic, infectious or autoimmune diseases, organ failure, hematological diseases or malignancies, no use of analgesic or anti-inflammatory drugs, corticosteroids, or immune modulating therapies. The study was carried out in accordance with the principles of the Declaration of Helsinki as revised in 2001 and approved by local ethics committee. All patients gave signed informed consent prior to inclusion in the study.

### 2.2. Clinical and Metabolic Variables

In all patients body mass index (BMI), waist circumference (WC), therapy for DM (oral hypoglycemic agents, insulin, combined treatment) and measured blood pressure, serum fasting glucose, glycated hemoglobin (HbA1c), creatinine, cholesterol, and triglyceride level, and urinary albumin excretion (UAE) were recorded. Hypertension was defined according to the 2013 European Society of Hypertension and the European Society of Cardiology criteria [20]. Hypercholesterolemia was defined as total serum cholesterol ≥ 5.18 mmol/L and hypertriglyceridemia as total serum triglyceride ≥ 1.7 mmol/L [21].

All the patients were asked on the presence of pain in the preceding month and pain intensity was measured on a 0–10 numerical rating scale (NRS). The presence of probable or definite NP was diagnosed according to the NP grading system [22].

### 2.3. Electrodiagnostic Assessment

The electrodiagnostic assessment was done with a four-channel Oxford Synergy electromyograph (Medelec Oxford, UK). An infrared lamp was used to maintain skin temperature > 31°C during all the tests. All the patients underwent sensory and motor NCS (band-pass filter 10–5000 Hz) of the lower limb nerves according to the American Association of Electrodiagnostic Medicine guideline [23] to explore the function of large nerve fibers. Amplitude of sensory nerve action potential (SNAP) and sensory nerve conduction velocity (SNCV) of the left sural nerve, and amplitude of compound muscle action potential (CMAP), motor nerve conduction velocity (MNCV), and minimal F-wave latency of the left peroneal nerve were recorded. SSR to supramaximal median nerve electrical stimulation was recorded (band-pass filter 0.1–100 Hz) from the left foot in all the patients.

### 2.4. Quantitative Sensory Testing

QST was performed by a trained examiner (F.M.) using a TSA-II NeuroSensory Analyzer (Medoc Ltd., Ramat Yishai, Israel). Thresholds were determined with the Method of Limits [24, 25]. Heat and cold stimuli were delivered through a 30 × 30 mm^2^ thermode attached to the skin of the dorsal surface of the left foot with a constant pressure. To determine the warm detection threshold (WDT) and the cold detection threshold (CDT), the skin was allowed to adapt to a temperature of 32°C for 5 min and then cooled down or warmed up linearly at a slow rate (1°C/s) until warm and cold sensation was perceived, at which moment the subject stopped the stimulus by pressing a button on a patient response unit. Warm and cold stimulation were repeated four times each and the mean of peak temperatures was considered threshold. Testing was preceded by detailed instructions to subjects and a demonstration test for each type of stimulus and performed in a designated, quiet room with no distractions [18, 26]. QST evaluation explored the function of small nerve fibers. In particular, WDT and CDT measure C and A-delta nerve fiber loss-of-function, respectively [27].

### 2.5. Blood Collection and Cytokines Assay

Blood samples were obtained in the nonfasting state at the same time of the day (i.e., 2 pm-3 pm) in all the patients. Glucose serum concentration at the time of blood sample was checked. After 10 min of rest in the supine position, blood samples were collected from the antecubital vein. Serum and plasma were immediately separated by centrifugation and stored in aliquots at −80°C until analysis. Serum concentration of TNF-*α* (n.v. < 15.6 pg/mL), IL-2 (n.v. < 31.2 pg/mL), IL-4 (n.v. < 31.2 pg/mL), IL-6 (n.v. < 3.12 pg/mL), and IL-10 (n.v. < 7.8 pg/mL) were assessed in duplicate by an enzyme-linked immunosorbent assay (ELISA) kit (Quantikine, R&D Systems, Minneapolis, USA), according to the manufacturer's protocol.

### 2.6. Statistical Analysis

All tests were carried out with the IBM SPSS version 20.0 statistical package. The normality of variable distribution was analyzed with the Skewness-Kurtosis test. Continuous variables were explored with ANOVA and post hoc *t*-test with Bonferroni's correction. Homogeneity of variance was analyzed with the Levene test. The data were transformed (logarithmic transformation) before submitting them to ANOVA in case of an inequality in the variances. The nonparametrical Mann-Whitney *U* test was applied in case the distribution was not normal. Pearson's *χ* ^2^ test with Yates' correction for continuity was applied to dichotomous variables. The correlation between the serum concentration of cytokines and electrodiagnostic parameters and other potential confounders (glucose serum concentration, age, BMI, sex, diabetes duration, and HbA1c) was explored with Spearman's *ρ* correlation coefficient. The variables, which turned out to be significant with Spearman's *ρ* correlation coefficient, were entered into multivariate regression analysis.  *p* < 0.05 (two-tailed) was taken as the significance threshold for all the tests.

## 3. Results

Clinical characteristics of the patients are reported in Table 1.

Large fiber neuropathy was found in 7 patients, small fiber neuropathy in 8, and neuropathy involving both large and small fibers in 11, while 6 patients had no neuropathy.

TNF-*α*, IL-2, and IL-4 were within normal values in all patients. IL-6 was raised in 14 patients (IL-6+ group) and normal in 18 patients (IL-6− group). IL-10 was raised in 5 patients (IL-10+ group) and reduced in 27 patients (IL-10− group).

BMI was significantly smaller in IL-10+ patients (25.2 ± 3.2 kg/m^2^) in comparison to the IL-10− group (30.5 ± 5.5 kg/m^2^; Mann-Whitney *U* test: *p* = 0.02; Table 1). HbA1c was significantly higher in the IL-6+ group (63 ± 14 mmol/mol) than in the IL-6− one (54 ± 12 mmol/mol; Mann-Whitney *U* test: *p* = 0.03; Table 1). The other demographic (age, sex), clinical (disease duration, therapy for DM, BMI, WC, hypertension, hypercholesterolemia, and hypertriglyceridemia), and metabolic variables (serum fasting glucose, HbA1c, creatinine, and UAE) did not significantly differ according to the presence or absence of raised IL-6 and IL-10 (Table 1).

Pain was reported by 26 patients, and NP was diagnosed in 18 of them. The number of patients with pain, the number of patients with NP, and the pain severity according to NRS did not differ in IL-6+ and IL-6− groups or in IL-10+ and IL-10− groups.

Among electrodiagnostic measures, SNAP amplitude was significantly smaller in IL-6+ group (4.4 ± 6.8 *μ*V) than in IL-6− group (9.0 ± 6.9 *μ*V, *p* = 0.033) and in IL-10+ group (1.6 ± 3.5 *μ*V) than in IL-10− group (8.2 ± 7.2 *μ*V, *p* = 0.032), CMAP amplitude was significantly smaller in IL-6+ group (3.2 ± 2.2 mV) than in IL-6− group (5.8 ± 3.7 mV, *p* = 0.030) and in IL-10+ group (1.0 ± 1.3 mV) than in IL-10− group (5.4 ± 3.2 mV, *p* = 0.003), MNCV was significantly reduced in IL-10+ group (31.8 ± 5.2 m/s) compared to IL-10− group (42.2 ± 6.1 ms, *p* = 0.009), and F-wave minimal latency was significantly longer in IL-10+ group (67.4 ± 13.5 ms) than in IL-10− group (49.3 ± 6.5 ms, *p* = 0.042), while the other NCV as well as the SSR measures did not significantly differ among groups (Table 2).

Glucose serum concentration at the time of blood sample, age, BMI, sex, diabetes duration, and HbA1c were not significantly correlated with IL-6 and IL-10 concentration.

**Figure 1:**
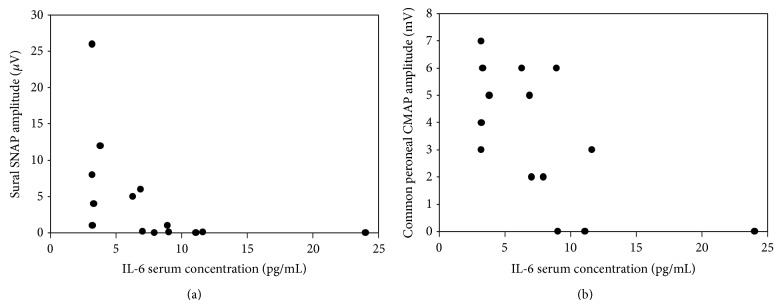
The correlation between the serum concentration of IL-6 and sural nerve sensory nerve action potential (SNAP) amplitude (Spearman's *ρ* correlation coefficient = −0.085, *p* < 0.001; panel (a)) and common peroneal nerve compound muscle action potential (CMAP) amplitude (Spearman's *ρ* correlation coefficient = −0.067, *p* = 0.009; panel (b)). For both correlations, Spearman's *ρ* correlation coefficient turned out to be significant.

The amplitude of sural nerve SNAP was negatively correlated with IL-6 concentration in patients with abnormal IL-6 values (Spearman's *ρ* correlation coefficient = −0.085, *p* < 0.001; Figure 1(a)). The amplitude of common peroneal CMAP amplitude was negatively correlated with IL-6 concentration in patients with increased IL-6 values (Spearman's *ρ* correlation coefficient = −0.067, *p* = 0.009; Figure 1(b)).

The correlation between IL-10 concentration and electrodiagnostic measures was not explored because of the small number of patients (*N* = 5) with increased values of this cytokine.

QST measures of small nerve fiber damage did not significantly differ when comparing IL-6+ and IL-6− groups or IL-10+ and IL-10− groups (Table 3).

Multivariate analysis was not performed because potential confounders did not turn out to significantly influence IL-6 or IL-10 concentration.

## 4. Discussion

The results of our study show that serum cytokines were abnormally raised in a group of patients with type 2 DM and that this finding was associated with DPN. Raised serum levels of IL-6 and IL-10 correlated with reduced amplitude of sural nerve SNAP and common peroneal nerve CMAP. Raised IL-10 serum level was associated with reduced common peroneal nerve MNCV and delayed minimal F-wave latency. No differences were found in pain characteristics, SSR and QST findings in relation to the presence of raised IL-6 and IL-10. These findings suggest that both cytokines may be related to axonal damage to large but not small nerve fibers and that IL-10 may also be associated with nerve demyelination. These cytokines do not seem to play a direct role in the pathogenesis of NP in our patients.

The present data add to previous evidence and offer a new perspective on the role of cytokines and other soluble immune mediators in the pathogenesis of DPN and help translating the bulk of data from animal models to the clinical setting in humans.

ILs play different roles in the immune system but they can be roughly divided into proinflammatory (TNF-*α*, IL-4, and IL-6) and anti-inflammatory ones (IL-2, IL-10), and some of them, which are termed neuropoietic, seem to have neuroprotective effects [2].

Serum IL-6 was raised in 44% of our patients and elevated IL-6 was significantly and inversely correlated with SNAP and CMAP amplitude. Taken together these data suggest a role of IL-6 in peripheral nerve axonal damage. Our findings are in accordance with a previous report showing that, among cytokines and inflammation markers, IL-6 was the most consistently associated with DPN [4, 14]. These data are also in keeping with the notion that IL-6 is a proinflammatory cytokine and may affect glial cells and neurons to set the pathological process of DPN in motion [2]. IL-6 is also a member of the so-called neuropoietic cytokine family [28], which reduces neurotoxicity in vitro and participates in neural development and has neurotrophic activity in vivo [29]. IL-6 was demonstrated to correct peripheral nerve dysfunction in experimental diabetes in rats [29] through correction of vascular endothelium dysfunction and increase of nerve blood flow via a mechanism that involves endothelium-derived hyperpolarizing factor [30]. Because of this contrasting experimental evidence and the absence of a follow-up in the present study, we cannot say whether IL-6 causes axonal nerve damage or it reflects compensatory or neuroprotective mechanisms.

Serum IL-10 was raised in 16% of patients and was associated with reduced SNAP and CMAP amplitude as well as with reduced MNCV and delayed minimal F-wave latency, suggesting that this cytokine might be associated with both axonal degeneration and demyelination. The mechanism of DPN is considered to be mainly axonal apart from diabetic patients with chronic inflammatory demyelinating polyneuropathy (CIDP), a condition that is more frequent in diabetic patients than in the general population. None of our patients satisfied the diagnostic criteria for CIDP [31], suggesting that some DPN patients may show demyelinating phenomena in addition to axonal ones without a clear diagnosis of a demyelinating polyneuropathy. Alternatively, the severity of axonal damage involving prevalently higher-velocity nerve fibers might have resulted in MNCV reduction in our patients. The number of patients with IL-10 abnormal values was however small and these results should be interpreted with caution. Different from MNCV, sural nerve SNCV did not significantly differ according to IL-10 levels.

IL-10 is considered an anti-inflammatory cytokine. Minocycline, a drug with anti-inflammatory and immunomodulatory effects, has been found to cause IL-10 to increase in parallel with the decrease of proinflammatory cytokines in a mice model of diabetic neuropathy [32]. We may speculate that the activation of IL-10 may represent a neuroprotective mechanism in response to severe peripheral nerve damage. The view of a compensatory mechanism is in keeping with previous evidence of raised serum heat shock protein 27, which has a role in neuroprotection, in patients with DPN [33], but a larger sample of patients and follow-up data are necessary to support it.

We could not document any abnormality in TNF-*α* in our patients. This finding was unexpected because this cytokine has been documented to contribute to the pathogenesis of type 2 DM complications including DPN [34], NP [8], and nerve degeneration [35]. However, the role of this cytokine is still controversial and our results are in accordance with a previous study, where TNF-*α* was not found to be associated with DPN [4]. Data from animal models documented that TNF-*α* was downregulated in the dorsal root ganglion in early stages of experimental diabetes, while the opposite occurred in later stages [36]. It may thus be hypothesized that one of the reasons of normal TNF-*α* in the present study might be the inclusion of patients with early or preclinical DPN, given that 19% had no neuropathy and involvement of small and large fiber was present in only 34% of them.

We could not find any association between pain and cytokine activation in our population of patients. This finding is in keeping with the absence of any correlation between serum cytokines and small nerve fiber damage as documented by SSRI and QST and with a previous report showing that thermal detection threshold and pain were not associated with serum immune mediators [4]. They are, however, in contrast with previous reports on other types of peripheral neuropathy, where patients with painful polyneuropathy show higher levels of serum cytokines in comparison to those with painless one [9, 15]. They also contrast those obtained in diabetic lumbosacral radiculoplexus neuropathy (DLRPN), where TNF-*α* was found to be elevated and to mirror the response to therapy [37]. A number of hypotheses may be suggested to explain our results. The first is that subclinical inflammation may only affect certain components of DPN, whereas others may be independent of immune activation [4]. The second is that the limited sample of cytokines tested in the present study did not allow thorough exploration of immune and inflammatory mechanisms involved in DPN-related NP [15]. The third is that the relatively low intensity of pain in our patients, probably resulting from no analgesic or anti-inflammatory treatment among the inclusion criteria of the study, might have biased our population towards low pain severity patients and this might be one of the reasons of the absence of any correlation with cytokine serum levels. The fourth one is that, despite the low pain intensity in our patients, some central sensitization mechanisms might prevail in the pathogenesis of NP in DPN [1, 7] in contrast to other types of diabetic neuropathies, such as DLRPN, where peripheral nerve microvasculitis has been suggested as the pathogenetic mechanism [37, 38]. Among clinical and metabolic measures, BMI was significantly smaller in patients with raised IL-10, and HbA1c was significantly higher in those with raised IL-6. However, demographic and metabolic variables were not significantly correlated with cytokine serum concentration. These findings together suggest that the severity of diabetes and metabolic derangement might be associated with cytokines activation [39], but the absence of any correlation with other metabolic variables impede further hypotheses.

Limitations of our study include the relatively small number of patients tested and the examination of serum levels of cytokines. The level of circulating cytokines might be influenced by a number of factors that we have tried to rule out by strict inclusion criteria, but confounding factors may not be completely ruled out. Exploring the cytokine level or their expression directly in nervous tissues such as in small fibers from skin biopsy or in nerve biopsy might offer data that are more tightly correlated with the clinical and neurophysiological findings, but these approaches would have been more invasive. Another limitation is that this study was cross-sectional, so it is not possible to separate risk or causative factors from merely coincidental associations or compensatory phenomena. Finally, we did not perform an extensive evaluation of autonomic functions that represent another feature of peripheral nerve damage in DM.

## 5. Conclusions

Exploring NCS and QST data, we have documented that, among the cytokines we tested, IL-6 and IL-10 were raised in some patients with DPN and correlated these abnormalities to axonal and in part demyelinating changes in large nerve fibers.

The present data might help understanding the pathogenesis of this complication of type 2 DM and to better target immune modulating treatments [2] for its prevention and treatment that to date represent an unsolved issue. The cross-sectional nature of the study did not allow understanding the extent to what these abnormalities contribute to nerve damage or represent compensatory or neuroprotective mechanisms. Future studies on larger populations of patients, with a follow-up, and including data from skin and/or nerve biopsies and CSF are needed to overcome the limitations of the present report.

## Figures and Tables

**Table 1:** Clinical characteristics of the patients.

	IL-6+ (*n* = 14)	IL-6− (*n* = 18)	IL-10+ (*n* = 5)	IL-10− (*n* = 27)
Age (years)	65.9 ± 9.2	62.4 ± 8.8	68.4 ± 9.4	63.1 ± 8.9
Sex (M/F)	8/6	9/9	4/1	13/14
Disease duration (years)	14.8 ± 8.3	15.2 ± 11.5	18.4 ± 9.5	14.4 ± 10.2
Therapy (oral/insulin/combined)	7/6/1	10/6/2	2/3/0	15/9/3
BMI (kg/m^2^)	30.2 ± 6.9	29.3 ± 4.3	25.2 ± 3.2^*^	30.5 ± 5.5
WC (cm)	108.4 ± 16.2	100.6 ± 10.5	97.6 ± 7.2	105.2 ± 14.3
Hypertension (yes/no)	9/5	11/7	4/1	18/9
Hypercholesterolemia (yes/no)	6/8	9/9	0/5	15/12
Hypertriglyceridemia (yes/no)	3/11	7/11	0/5	10/17
Pain (yes/no)	12/2	14/4	4/1	22/5
Pain severity (0–10 NRS)	2.1 ± 2.3	2.5 ± 2.3	1.9 ± 1.2	2.4 ± 2.4
Serum fasting glucose (mmol/L)	9.0 ± 2.7	9.0 ± 2.8	8.4 ± 2.6	9.3 ± 2.7
HbA1c (mmol/mol)	63 ± 14^*^	54 ± 12	56 ± 8	58 ± 16
Creatinine (*µ*mol/L)	97.2 ± 53.0	97.4 ± 61.9	106.1 ± 79.6	97.2 ± 53.0
UAE (mg/L)	288 ± 193	210 ± 204	253 ± 235	314 ± 255

BMI: body mass index, WC: waist circumference, NRS: numerical rating scale; HbA1c: glycated hemoglobin, and UAE: urinary albumin excretion from spot urine sample.  ^*^ *p* < 0.05.

**Table 2:** Electrodiagnostic measures.

	IL-6+ (*n* = 14)	IL-6− (*n* = 18)	*p*	IL-10+ (*n* = 5)	IL-10− (*n* = 27)	*p*
Sural nerve						
SNAP amplitude (*µ*V)	4.4 ± 6.8, 6.6	9.0 ± 6.9, 10.5	0.033	1.6 ± 3.5, 0.8	8.2 ± 7.2, 10.0	0.032
SNCV (m/s)	47.5 ± 9.8, 47.2	45.3 ± 8.5, 43.8	n.s.	49.0 ± 10.9, 47.0	45.8 ± 8.9, 45.3	n.s.
Common peroneal nerve						
CMAP amplitude (mV)	3.2 ± 2.2, 5.3	5.8 ± 3.7, 7.4	0.030	1.0 ± 1.3, 0.5	5.4 ± 3.2, 6.5	0.003
MNCV (m/s)	40.0 ± 6.5, 43.9	42.1 ± 7.0, 44.2	n.s	31.8 ± 5.2, 34.0	42.2 ± 6.1, 44.6	0.009
F-wave minimal latency (ms)	54.7 ± 10.5, 52.0	48.5 ± 6.8, 49.2	n.s.	67.4 ± 13.5, 65.2	49.3 ± 6.5, 49.2	0.042
SSR						
Latency (s)	1.4 ± 0.3, 1.3	1.5 ± 0.2, 1.4	n.s.	1.5 ± 0.1, 1.6	1.5 ± 0.3, 1.4	n.s.
Amplitude (*µ*V)	0.9 ± 0.6, 0.8	1.0 ± 0.7, 1.0	n.s.	1.0 ± 0.3, 0.9	1.0 ± 0.7, 1.0	n.s.

Data are presented as mean ± SD, median. *p* values are from Mann-Whitney *U* test. SNAP: sensory nerve action potential, SNCV: sensory nerve conduction velocity, CMAP: compound muscle action potential, MNCV: motor nerve conduction velocity, and SSR: sympathetic skin response.

**Table 3:** Quantitative sensory testing measures.

	IL-6+ (*n* = 14)	IL-6− (*n* = 18)	*p *	IL-10+ (*n* = 5)	IL-10− (*n* = 27)	*p*
WDT (°C)	42.8 ± 4.9, 42.9	42.1 ± 4.5, 41.4	n.s.	44.0 ± 5.2, 43.7	42.0 ± 4.4, 41.4	n.s.
CDT (°C)	20.9 ± 8.9, 25.9	20.7 ± 9.3, 22.7	n.s.	19.7 ± 8.7, 23.8	21.0 ± 9.2, 24.6	n.s.

Data are presented as mean ± SD, median. *p* values are from Mann-Whitney *U* test. WDT: warm detection threshold, CDT: cold detection threshold.
